# Safeguarding implementation elements in a national school mental health intervention: implementers’ perspectives

**DOI:** 10.3389/fpubh.2026.1895117

**Published:** 2026-10-28

**Authors:** Loreto Leiva, Gloria Zavala-Villalón, Fernanda Sánchez-Vilaró

**Affiliations:** Department of Psychology, University of Chile, Santiago, Chile

**Keywords:** consolidated framework, implementation research, mental health, qualitative research, school mental health

## Abstract

**Objective:**

The purpose of this study was to identify and describe implementation determinants that are sensitive when undertaking a school mental health intervention and that require particular attention during implementation.

**Methods:**

A multisite qualitative study was conducted using a deductive analytical approach. Thirty team members implementing a national preventive school mental health program were interviewed. The interviews were analyzed using content analysis based on pre-established categories from the Consolidated Framework for Implementation Research (CFIR).

**Results:**

The findings highlight several implementation conditions requiring particular attention, including resources and time; training, feedback and monitoring; structural alignment across organizational levels; school over-intervention; and the recognition of adaptations as plausible elements within an intervention.

**Discussion:**

This research provides empirical insights regarding the implementation factors of SMH programs within authentic school settings. Furthermore, the qualitative depth achieved through interviews with both implementers and coordinators allows a multi-level understanding of the factors influencing long-term sustainability and the intervention’s ecological validity.

## 1 Introduction

Faced with the proliferation of mental health problems affecting children and adolescents, School Mental Health (SMH) interventions have been recognized as a key strategy to address this challenge, demonstrating promising results globally (1, 2). They are currently among the most widely used tools worldwide and can be implemented sustainably in diverse cultural and educational models (3).

SMH interventions positively impact the risk trajectories of children and adolescents (3, 4), are cost-effective (5), contribute to academic performance (6–9) and are associated with improvements in emotional and behavioral domains (3, 10). Evidence suggests that school-based mental health frameworks provide an environment for health and well-being and create spaces that protect children and adolescents from risk factors (11, 12).

Although these interventions are widely recognized, the results may differ from expectations when they are implemented in the community. Even the same standardized SMH intervention can be executed differently with varying levels of fidelity, leading to heterogeneous outcomes across diverse local contexts (13). This illustrates the gap between what is expected from effective interventions in controlled contexts and what actually occurs in real-world implementation settings (14, 15). When interventions are carried out in the “real world,” they may not retain the same level of precision, reflecting adaptations and/or modifications. This is not inherently problematic, as the goal of implementation is not fidelity in and of itself, but rather the value the intervention generates; that is, the relationship between its benefits and the resources or costs involved (16).

While it is expected that SMH interventions will remain faithful to the original design and be executed with the same quality with which they were designed and tested, it should be considered that those who implement interventions often make modifications (deliberate or unintentional) that can influence implementation and intervention outcomes (17, 18). The literature suggests that adaptations are an inherent and inevitable feature of the implementation process, rather than an exception (19, 20), allowing interventions to respond to the specific needs of a particular population, making them culturally relevant and situated (21, 22).

Furthermore, indeed, adaptation helps ensure that an intervention fits the local context and is relevant to those who receive it, as an intervention that is “perfectly faithful” but does not align with the culture, resources, or needs of a community ultimately has limited value because it is either not used or fails to achieve the expected outcomes (16).

Evidence has shown that adaptation is not only favorable for implementation but also necessary (1, 23, 24), providing ecological relevance to the intervention and making it culturally relevant to the population where it is being implemented (22, 25, 26).

This perspective challenges the traditional emphasis on strict adherence to original protocols and suggests that thoughtful adaptations may be necessary to ensure that an intervention fits the context and remains sustainable. Adaptation has been described as the mechanism that allows science to “land” in the real world. Without it, evidence-based interventions risk becoming irrelevant or impossible to implement in settings that differ from controlled clinical trials (16).

Thus, assuming that adaptations are an unavoidable part of executing an intervention, literature highlights the importance of finding the right balance between implementation fidelity and adaptation to local contexts (17). This balance requires distinguishing between the essential functions an intervention is intended to fulfill and the different ways these functions may be delivered across different contexts. From this perspective, safeguarding implementation does not necessarily mean maintaining a fixed way of delivering an intervention; rather, it involves preserving the conditions necessary for its intended functions to be maintained, while allowing flexibility in how these functions are carried out at the local level (16, 18, 27). Understanding these conditions is a key contribution of implementation science, particularly in school mental health, where interventions are implemented within complex and diverse organizational contexts. In this regard, implementation science provides useful frameworks for examining the contextual, organizational, and individual factors that may facilitate or constrain the delivery of an intervention as intended.

It is common for interventions with established efficacy in controlled trials to fail to achieve the expected results in schools (1, 28). Implementation Science (IS) sheds light on why the effects can vary so widely when the same design is used across different contexts.

IS enables the investigation of how interventions are delivered in practice, acknowledging that the conditions of controlled designs are not always reflected in real-world implementation (29). The impact of implementation is so significant that it is considered the second-most important variable for achieving results (1, 28).

It has been demonstrated that the implementation of interventions reaches its full potential when it follows the planned instructions and steps for their application while also adapting to be ecologically relevant (16). In other words, implementation adapts to the needs and conditions of the setting or population in which the intervention will be implemented (1, 25, 30).

Specifically, in school mental health, adaptations that respect essential design elements benefit students while allowing those who perform the intervention to improve their implementation (17). Thus, to implement interventions successfully, elements that are part of (a) implementation conditions and beneficiaries, (b) implementers, and (c) the implementation itself should be considered.

### 1.1 Elements associated with implementation conditions and beneficiaries

Culturally blind interventions erode results because they fail to stimulate community participation (31). However, excess cultural centralization does not guarantee results, even if it may be attractive to beneficiaries (21).

Therefore, current research emphasizes the importance of assessing pre-implementation conditions by studying execution locations to create designs tailored to the needs, context, and base program. This includes identifying available resources (both material and human), the target population to be intervened (primary and secondary beneficiaries), the leadership that supports implementation (in the internal and external context of implementation), the Implementation Climate (receptivity and propensity for change), and the knowledge accessed (training), among other factors to consider for generating culturally relevant interventions (2, 19, 22, 25, 31).

### 1.2 Elements associated with implementers

The literature highlights the role of qualified personnel (29). In the case of SMH interventions, this does not mean that all implementers need to be mental health professionals, as good results have been reported with various types of professionals in charge, provided they are properly trained (32) and frequently evaluated in their practice (33).

The team responsible for delivering the intervention needs to be prepared for change and innovation, demonstrating “adaptive expertise,” understood as the ability of professionals to apply their knowledge flexibly and creatively in new, complex, or ambiguous situations, such as mental health in school settings (34). To ensure that the team is adequately prepared for implementation, it is necessary to consider their perspectives, provide opportunities for reflection and support, strengthen group motivation and initiative, and incorporate their feedback into subsequent versions of the intervention (32, 34).

### 1.3 Elements associated with implementation

It is essential to understand that implementation is dynamic; it should be conceived as a process of change and requires constant adaptation to be sustainable (35). Thus, for proper execution, interventions need to be designed that allow modifications and provide options for achieving objectives (1). Engell et al. (27) propose a “common elements” approach that enables implementation to remain dynamic and adaptable without losing its “core essence.”

In addition, intervention design should include continuous evaluation of implementation processes and implementers (36), with feedback on emerging challenges used to inform subsequent iterations of the intervention (33).

Since various elements can influence the successful implementation of an intervention, the following question arises: What implementation elements should be safeguarded when implementing a mental health intervention in schools? This question can be addressed through the operationalization of the Consolidated Framework for Implementation Research (CFIR) (37, 38).

The CFIR is a multi-theoretical framework for IS that assembles various factors that the literature has considered relevant in implementation. It is applicable to a wide range of interventions, research designs, and environments (39), such as schools (40–42). The framework organizes potential implementation determinants into five interconnected domains, providing a structured way to examine the contextual, organizational, and individual factors that may shape implementation. In the context of SMH, examining these factors can help identify the conditions that facilitate or constrain the implementation of an intervention, including the capacity of implementation teams and organizations to adapt its delivery while preserving its intended functions.

This framework organizes potential implementation factors into five interconnected domains: Intervention characteristics, inner setting, outer setting, individual characteristics and the implementation process. These domains offer a systematic approach to identifying factors that may influence implementation outcomes. These dimensions are relevant to achieving optimal expected outcomes in interventions and should therefore be safeguarded during implementation (38). This framework can inform the identification and description of implementation conditions that are particularly sensitive to execution in SMH interventions. Identifying these factors is a step toward understanding which elements should be safeguarded to ensure proper execution, given the need to increase knowledge of the support required to optimize interventions in SMH for children and adolescents.

Given the above, there is a need to better understand which implementation conditions are perceived by implementers as particularly important for maintaining the integrity and contextual fit of SMH interventions. This study will focus on the Chilean program Skills for Life (SfL; Habilidades para la Vida, or HpV for its acronym in Spanish). This program was selected because it is a large-scale school mental health initiative targeting the educational community and is implemented in more than a quarter of schools nationwide (43). Although it is one of the largest school mental health initiatives worldwide (3), the program faces common challenges associated with routine implementation across diverse educational settings, which require differentiated implementation strategies. Guided by the CFIR framework, this study aims to identify and describe implementation determinants that implementers perceive as important to safeguard during the implementation of a national SMH intervention, with attention to the elements necessary to maintain its integrity and contextual fit. Thereby contributing to the program’s long-term sustainability through enduring school-based practice that consolidates the intervention’s achievements.

## 2 Materials and methods

### 2.1 Procedure

The study involved members of the implementation teams (implementers) of the Skills for Life (SfL) program. SfL is a school mental health program implemented nationwide from elementary school (SfL I and SfL II) through high school (SfL III). Implementers deliver promotional and preventive mental health strategies based on the three levels recommended by the World Health Organization: (a) universal promotion or prevention, which strengthens conditions that support positive mental health; (b) selective prevention, which identifies students at risk of developing mental health problems and provides preventive interventions (e.g., workshops); and (c) indicated prevention, in which students at higher risk are referred to external mental health services (43).

In Chile, SMH is addressed through an intersectoral structure involving the national government, municipalities, schools, and the health system. At the national level, the Ministry of Health, the Ministry of Education and the National Board of School Assistance and Scholarships play complementary roles in developing policies, guidelines, and programs related to mental health promotion and prevention in educational settings.

At the local level, municipalities coordinate and implement school mental health initiatives through teams that work directly with schools. These teams collaborate with school leadership and other members of the educational community. When students require support beyond the scope of school-based promotion and prevention, coordination with the health system enables referrals to primary care or specialized mental health services.

Within this structure, the SfL program is implemented at the local level through teams that work directly in schools to deliver mental health promotion and prevention strategies. Specifically, this study focused on the activities and strategies used by implementation teams in elementary schools (SfL I, targeting children aged 6–8 years, and SfL II, targeting those aged 11–13 years), with an emphasis on universal and selective prevention in SMH. The universal prevention strategy involves activities targeting parents and teachers, whereas the selective prevention strategy targets students and is delivered throughout the school year at each participating school. The intervention consists of 10 sessions with students, 3 sessions with their parents, and 2 sessions with their teachers. These activities aim to alter the trajectory of identified risk by addressing behaviors early that could develop into future mental health disorders (43).

Implementation teams are organized at the municipal level and are composed primarily of psychologists, social workers, and other social science professionals. They are directly responsible for implementing the model proposed by the SfL program at schools. To support program delivery, implementers receive training and technical support. This training covers the program’s foundations and components, its mental health promotion and prevention strategies, and the use of the instruments and methods established for student assessment and monitoring. Implementers also receive guidance on the procedures for planning, delivering, and monitoring program activities. In addition, technical support from the national coordination team provides ongoing assistance during implementation and guides implementers in applying the model in accordance with its guidelines and technical requirements (43).

### 2.2 Study design

We employed a multisite qualitative research approach, guided by a deductive analytical focus driven by the domains and dimensions of the CFIR (37). Although an updated version of CFIR (38) is now available, we retained the original framework because the study, including its design, fieldwork and analysis, was conducted between 2022 and 2024, and the *a priori* coding framework had already been established at the start of the study. Modifying the framework midway through the analysis would have compromised the consistency and comparability of the coding process. Therefore, the coding scheme, construct definitions, and analytical categories were established and applied using the original framework (Supplementary material 1 provides a crosswalk between both versions).

We followed the Consolidated Criteria for Reporting Qualitative Research (COREQ) guidelines to ensure transparency and rigor in reporting (44).

### 2.3 Research team and reflexivity

Two researchers (LL and GZV) conducted the data collection and analysis, both of whom hold PhDs in Psychology and have extensive experience in mixed-methods and qualitative research, particularly in the field of school mental health. Both are university-based academics with in-depth knowledge of the SfL program. No prior relationship existed between the researchers and the participants. Before the interviews, participants were informed of the researchers’ credentials and of the study’s primary aim. At the beginning of each session, time was allocated to address any questions regarding the researchers’ backgrounds or the study objectives.

### 2.4 Study participants and sampling

We considered members of the executing teams (implementers) and coordinators of the national SfL from educational communities where the program was implemented. They were selected through purposive sampling to ensure a diverse representation of roles and geographic contexts within the SfL program. A total of 30 individuals participated, including 19 implementers, seven community coordinators, and four regional coordinators.

Implementers are responsible for delivering the program’s promotional and preventive strategies. They conduct workshops for children, parents, and teachers, and carry out the program’s established universal promotion activities. Community coordinators supervise implementation teams within each municipality and serve as a link between the teams, schools, and local government. Finally, regional coordinators oversee program management across municipalities, but do not deliver sessions. These participants represented educational communities across four regions of Chile, selected for their diverse geographic and demographic characteristics to capture a wide range of implementation experiences.

We contacted the potential participants via institutional email, followed by telephone calls to explain the study’s scope and invite them to participate. This purposive approach enabled the inclusion of key actors directly involved in implementing selective preventive interventions in basic education cycles. Details of the participants and their characteristics are presented in Table 1.

**Table 1:** Sample characteristics.

Characteristics	*N*	%
Sex		
Female	22	73.3%
Male	8	26.3%
Years working in the program		
Less than 1 year	1	3.3%
Between 1 and 3 years	12	40%
Between 3 and 5 years	9	30%
More than 5 years	8	26.6%
Role		
Implementers	19	63.3%
Community coordinators	7	23.3%
Regional coordinators	4	13.3%
Professional backgrounds		
Psychologist	12	40%
Social Worker	9	30%
Education Professional	1	3.3%
Other*	8	26.6%
Employment status		
Employee (employment contract)	18	60%
Independent contractor (fee-for-service)	12	40%

*Includes one anthropologist, two family counselors and five participants whose profession was not reported.

### 2.5 Data collection

We conducted semi-structured interviews based on the CFIR framework’s domains [e.g., Abry et al. (45)]. To ensure the instrument’s validity and clarity, two implementation science experts reviewed the interview guide (Supplementary material 2), which was then pilot tested with an implementer outside the final sample. Because participants were geographically dispersed, we conducted all interviews online via video conferencing platforms (Zoom and Teams), with participants joining from private spaces within their respective workplaces.

The interviews lasted 40–60 min, were audio-recorded and subsequently transcribed verbatim for analysis. We continued data collection until no new information emerged. This process was supported by monitoring the frequency distribution of mentions during the preliminary analysis. Following the analysis, the final research findings were shared with the participants to ensure the accuracy of the interpretations (participant checking).

### 2.6 Data analysis

We used qualitative content analysis with pre-established categories (46) based on the CFIR framework. To ensure the rigor and reliability of the findings, two researchers (GZV and FSV) independently coded the transcripts using Atlas.ti 9 software. Any discrepancies during the coding process were resolved through iterative discussion and consensus. When discrepancies persisted, a third researcher (LL) intervened to resolve the disagreement. Since coding discrepancies were resolved iteratively through discussion rather than by independently comparing codes, it was not possible to retrospectively calculate a quantitative measure of inter-coder agreement (e.g., Cohen’s kappa). This consensus-based approach is a recognized alternative to independent double coding in qualitative research (47).

We first performed a descriptive quantitative analysis of the CFIR model domains. Statements associated with the CFIR dimensions across the five established domains served as the units of analysis. Since the interview guide was structured around the CFIR domains, the number of coded units within each dimension reflects the instrument’s structure. Therefore, the findings cannot be interpreted as purely emergent categories.

For the analysis, we prioritized the most salient implementation elements using a salience criterion based on three conditions. First, an element had to fall within the upper 50% of the cumulative distribution of coded units, providing an initial indicator of coding density. Second, it had to be mentioned by at least two of the three participant groups, indicating that it was not specific to a single professional role. Given the unequal size of the participant groups, we also considered the number of individual participants who mentioned each element, rather than relying solely on the number of coded excerpts. Third, participants had to link the element to observed consequences for program implementation when the corresponding condition was absent, insufficient, or modified. To assess this criterion, we identified accounts describing concrete consequences for implementation (e.g., quotes 5, 9, and 17). Elements that met all three conditions were considered salient implementation elements and interpreted as elements that may warrant safeguarding.

We categorized the salient dimensions into three overarching themes. The identified dimensions were separated into three groups associated with implementation elements: (a) elements associated with implementation conditions and beneficiaries, (b) elements associated with implementers, and (c) elements associated with the implementation. This analytical structure enabled the integration of the findings into a coherent description of the participants perceived as necessary for safeguarding the intervention.

### 2.7 Ethical considerations

Our study was supported and authorized by the National Board of the Student Health Program and the National Coordination Agency of the SfL program and was approved by the Ethics Committee of the Faculty of Social Sciences, University of Chile. Participation was strictly voluntary, and participants provided informed consent. All personal identifiers were removed from the transcripts, and data were anonymized using alphanumeric codes. All digital files were stored in encrypted, password-protected folders, accessible only to the research team. Participants were informed of their right to withdraw from the study at any stage without any negative consequences.

## 3 Results

Using the domains and dimensions described by the CFIR model (37, 38), 30 semistructured interviews conducted with implementers and coordinators of the SfL program were analyzed to identify and describe implementation-relevant factors associated with implementation elements of this SMH intervention.

To prioritize the most prominent factors described by participants, only those dimensions present in at least two of the interviewed groups and within the top 50% of total citations were selected for detailed analysis. This criterion has been used in previous studies [e.g., Engell e al., (27)] and does not imply that domains outside the top 50% are peripheral elements.

The dimensions with the highest frequency were Readiness for Implementation, Adaptability, Reflection and Evaluation, and Implementation Climate. The dimensions of Needs and Resources of the individuals served by the organization and External Policy and Incentives also had a high number of citations. The six dimensions identified together accounted for 51.72% of the total citations (Table 2). The region-level data did not differ among implementers, community coordinators, or regional coordinators; the same six dimensions were among the most cited by all participants (details on the number of citations are provided in Supplementary material 3).

**Table 2:** Total citation counts by domain.

Domain (according to CFIR model)	Dimension (according to CFIR model)	Number of citations	Relative frequency (%)	Cumulative frequency (%)
Inner setting	Readiness for implementation	192	13.24	13.24
Intervention characteristics	Adaptability	150	10.34	23.58
Process	Reflecting and evaluating	120	8.28	31.86
Inner setting	Implementation climate	116	8.00	39.86
Outer setting	Patient needs and resources of individuals served by the organization	87	6.00	45.86
Outer setting	External policy and incentives	85	5.86	51.72

The dimensions identified were classified into the following categories: (a) elements associated with implementation conditions and their beneficiaries, (b) elements associated with implementers, and (c) elements associated with the implementation. This approach identified the elements associated with each condition that were important for the implementation.

Table 3 summarizes the specific elements identified within each category and their corresponding CFIR dimensions. The following sections describe these elements in more detail and provide illustrative quotes from participants.

**Table 3:** Characteristics of key elements according to the analysis using CFIR.

Elements	CFIR dimensions	Main findings: what to safeguard
Elements associated with implementation conditions and beneficiaries	Readiness for implementation: resources	Having infrastructure, materials and time for intervention implementation. Establishing adequate coordination with schools and managing workload. Accessing information and knowledge, expressed through relevant and timely training.
	Implementation climate	Support and involvement of school leadership teams. Municipal authorities’ support for program viability. Engagement of teachers in implementation. Avoiding over-intervention in schools.
	Patient needs and resources of individuals served by the organization	Having tools to address emerging issues required by the context. Flexibilizing intervention based on contextual needs.
	Foreign policy and incentives	Safeguarding decentralization in program design and variability in its execution, seeking alignment between organizational structures. Ensuring that the rotation of executives does not entail changes in the implementation of the intervention.
Elements associated with implementers	Readiness for implementation: leadership preparation	Having clear leadership from community coordinators, both in terms of supporting and validating team members and in managing the overall process.
Elements associated with implementation	Adaptability	Adapting aspects of the intervention to align with context-specific needs that are culturally relevant, without compromising its fidelity.
	Reflecting and evaluating	Establishing formal and frequent instances of reflection and evaluation. Having timely formal feedback in response to evaluations.

### 3.1 Elements associated with implementation conditions and beneficiaries

The dimensions included in this category are Readiness for Implementation, Implementation Climate, Needs and Resources of Individuals Served by the Organization, and External Policy and Incentives.

The first dimension, Readiness for Implementation, involves tangible and immediate indicators of the organization’s commitment to the execution of the intervention (e.g., available resources: time, money and space). Participants frequently identified access to adequate physical spaces and material resources as a primary facilitator for program delivery (e.g., computers and Internet connections) (Table 4, Quote 1).

**Table 4:** Textual quotations from interviews.

Quote N°	
1	“… schools provide us with spaces, some of which are good and some not so good. For example, when they give us the cafeterias, that’s very spacious, it’s very large to work with the kids, so it’s distracting, there’s a smell of food sometimes, so it’s unpleasant for the kids… with the smell of fried fish… others provide libraries, but it’s also complex because they say we cannot talk loudly, we cannot speak loudly, so well…”
2	“… yes, materials are purchased, but today we talked about the material, printers, computers; let us say we have only one printer that sometimes works very poorly, and there are 12 of us, and the coordinator said today that it could not be done, that there was a problem because the corporation does not allow it because the money that arrives comes at the end, you cannot buy, for example, a printer, I do not know why, it’s like… a law, I have no idea what it’s like, but you can only buy material that will be consumed.
3	“Yes, the program provides manuals, but they are very brief… now we request training, that’s important. Of course, we take advantage of the network day we have, and we are there, but for other topics or with more information… We send a list of training sessions according to our team’s needs, so let us hope…”
4	“(the implementation climate) will depend on the school because here, for example, we work very well with the principal. There is smooth communication; she informs us, we inform her when it’s not possible, or something like that. She asks us, etc., as if she incorporates us; that would be a characteristic here. She makes us a part of…”
5	“(to the principal) She did not like it, did not accept the program, did not find it good. She used to be the coordinator for SfL before and did not like the program when she became the principal. She would always leave us stranded. It was like when we went there, and there was nothing, no teachers, or no facilities to actually do things. We would schedule something, it could not be executed, and they would not let us go through with some things; other times, yes. So, we were at the mercy of how she was. When she wasn’t there, the inspector was there, and everything worked well. So, in the end, it was that person…”
6	“There are other teachers who get much more involved and ask about how the progress is. So, there’s also a personal interest from the teacher about what’s happening. There’s a bit of both. I could not say that everyone demands this, but it is a constant over the years. And it depends a lot on the connection. I think the connection one establishes has a significant impact. The principal is fundamental. If the principal does not accept the intervention or does not find it meaningful, ultimately, he’s the one who creates the spaces.”
7	“The municipality supports all program activities… when there are massive activities, we coordinate with the city hall, for example, because the city hall is the one in charge (…) the mayor says ‘I’m the one who matters here,’ and he’s the one who hires. The mayor can cancel the activity; everyone else may be there, but if he cannot, he cancels the activity.
8	“Some teachers also do not understand much because they always complain to me that they are over-involved…”
9	“…from my experience in the first year of implementation, there was a school, and they were incredibly over-involved. They had the health center almost every day, they had different networks, they had their own socioemotional program, so they really, well, I cannot take responsibility for what happened before, but I do not know what was offered to them as a program at that time because clearly, they could not adopt this program due to their schedules. So, they resigned at the end of the year, saying, ‘Honestly, we do not have the time, and thank you for what you did for us, but there is no more time to offer, so we are sorry.’”
10	“… the complicated part is communicating with the parents when a specific issue arises in a session, and we investigate and detect that there may be abuse. So, being able to approach those parents who do not understand us, making visits, the referral does not happen, so it’s very complex… deploying a lot of things that are difficult. But we are in discussions; in fact, now, towards the end of the year, we already have to evaluate another plan. In fact, we might create a manual or the appointments, we are doing appointments with parents in Creole, so with the date well marked that day, we will try to get a person who speaks Creole at the school to accompany us to the interview. But it’s very difficult, due to human resources, it’s very difficult…”
11	“In this particular commune, I believe that over the last 3 years, there has been a rotation of directors, and that is important. Maybe there is a lot of work done with the teachers, with the children, building relationships, and there is a change in leadership that might have a different focus, something more structured, less permeable. So, you have to do all the work as an introduction again, lobby, like starting everything over, so that also slows things down. I think the important thing is how you deal with that, that’s the important thing, see what happens, face it (…) But yes, indeed, I believe that the disposition of the director influences, as everything starts from there, from the top down.”
12	“I think the kids feel like they are part of it, I feel like I’m part of it too. As I said, our leaders are quite close, and I think that is also appreciated. There is flexibility; we are on an hourly basis, but if someone has a license, well, ‘you are sick, go ahead, take your days,’ in that sense, there is a lot of understanding.”
13	“So, the goal is respected because each session has a goal, but you still play around with it. For example, I tend to focus much more on the connection than on the methodology, so they are mainly… the sessions are more about recognizing, making it more fun, building connections, and a good connection allows you to progress and generate a greater impact.”
14	“In rural schools, they are always welcome, and any intervention you do is well received by the teachers. In fact, teachers ask, ‘HPV lady, when are you going to do a workshop?’ or ‘We have a parent meeting next week, can you bring a topic on abuse?’ for example. So, the reality is different, and therefore, the affection you have for rural schools is different from the larger ones.”
15	“We do it informally (instances of reflecting on practices and interventions). Certainly, when we meet, we talk like ‘What are you doing?’ They ask me ‘How did you do that if you have this workshop, if you have support hours?’ So, that’s informal, and we have technical meetings every 2 weeks, where we also discuss…”
16	“Like these evaluation sessions at the end of the first semester and end of the second semester in December, those are structured. It’s like each person in charge knows that they have to make a presentation with certain content, and it’s structured. After the presentation, there’s time for reflection on that.”
17	“…from all that one delivers, nothing… as I told you, there is no feedback, nothing is provided in terms of information, incentive, reinforcement, whether positive or negative, even constructive criticism. Everything serves to improve, but here it’s like we see it among ourselves.

However, this may not be entirely possible, as the intervention studied only included expensive consumable materials, despite the need for other resources to operate excellently (Quote 2).

There is also the challenge of limited time for carrying out activities, either due to high workloads or insufficient human resources. The lack of both physical structure and the necessary time for intervention would impact its functioning, development and success.

Regarding access to information and knowledge, there is a recognized need for better training. Implementers perceived a gap between the theoretical guidelines and the practical demands of diverse school contexts (Quote 3).

The second dimension, Implementation Climate, is defined as the shared receptivity and absorption capacity for change among those involved in the intervention, along with the associated reward within the organization. It was observed that support and involvement of school leadership teams are necessary to foster an appropriate Implementation Climate for the studied intervention. This would enable better communication with the institutions and legitimize the program, reducing potential barriers to coordinating work with teachers (Quote 4). This approach is particularly relevant because when school leadership teams are not receptive or do not engage with the program, a number of coordination and integration difficulties arise during implementation in schools (Quote 5).

The involvement of teachers in implementing interventions is also essential, and establishing a strong relationship between implementers and teachers is crucial for this. The role of school leadership team members in building this relationship is also emphasized (Quote 6). Finally, the support of the main municipal authority is highlighted, as it makes the program feasible and visible in the community (Quote 7).

A specific challenge identified in urban settings was ‘over-intervention’, where the presence of multiple programs leads to a perceived saturation and diminished commitment from school staff. Many schools, especially urban schools with a large number of students, are exposed to more than one program. In these cases, institutions were observed to value the SfL program less, leading to less dedication and time from leaders and teachers, and less effort to make the necessary adjustments to accommodate the program (Quote 8). This implies that the program requires tailoring to the needs of the schools to ensure its continuity and viability (Quote 9).

Third, associated with the implementation conditions and beneficiaries, there are “needs and resources of individuals served by the organization.” This refers to understanding and prioritizing beneficiaries’ requirements, as well as the barriers and facilitators to meeting those needs. The focus here is on users, their needs, and the resources they have for implementing the intervention.

With respect to needs, tools are required to address emerging issues. In the specific case of the intervention studied, participants emphasized the need for the program to evolve and incorporate emerging social themes, such as sexual diversity and multiculturalism, to maintain contextual relevance (Quote 10) This highlights the need for continuous evaluation of the intervention content, which can address emerging issues that may directly or indirectly affect participants’ mental health. This translates into an explicit request to incorporate this content in the intervention through the training of implementers.

Finally, the central axis of the dimension of External Policy and Incentives, which refers to external strategies for disseminating innovations, regulations, mandates, guidelines, recommendations and policies (governmental and civil), is the decentralization of the program and the need for alignment among different organizational structures.

Given the program’s structure and decentralization from the central government to local governments (e.g., municipalities), aligning the program with community spaces is hindered because the units to which it could be attached (e.g., health, education, corporations) vary in scope and impact. The analysis revealed that political and administrative volatility in local governments often leads to leadership turnover, which is perceived as a significant barrier to the continuity of program objectives. These administrative changes can potentially impact implementation by altering plans and priorities in establishments directly affiliated with municipalities (Quote 11).

Overall, participants indicated that ensuring the conditions surrounding program delivery and its beneficiaries requires: (a) sufficient material, human, and time resources; (b) a receptive Implementation Climate, supported by collaborative work with school leadership teams, teaching staff, and municipal authorities; (c) mechanisms to incorporate beneficiaries’ emerging needs into the program content; and (d) stability and coordination across the structures responsible for the program, particularly in contexts with high staff turnover.

### 3.2 Elements associated with implementers

Only the dimension of Readiness for Implementation stands out in this category, focusing on preparation for leadership (e.g., participation, responsibility and commitment of leaders in the implementation). The role of community coordination was identified and emphasized in the studied SMH intervention. Participants emphasized that coordination needed to be clearly identifiable and provide support to team members when necessary. Characteristics such as collaborative leadership are valued over hierarchies (Quote 12). This highlights the need for leaders who are recognized for the support and contribution they can provide rather than for the sanctions or control they might enforce.

Although the evidence suggests that individual qualifications, training, and implementers’ ability to adapt are fundamental (32–34), implementers focused their responses more on the leadership and coordination they received than on their own characteristics. Issues related to implementer training and preparation focused mainly on readiness for implementation, both as a broader condition for implementation and as a characteristic of the implementers themselves. This suggests that, from the perspective of those delivering the program, structural and relational support (e.g., clear and collaborative leadership that values people) was seen as more fundamental and relevant than individual professional characteristics.

### 3.3 Elements associated with the implementation

The dimensions of Adaptability, Reflection and Evaluation are deemed relevant in this category. Adaptability refers to the degree to which an intervention can be adjusted, refined, or reinvented to meet local needs. This approach was perceived as essential for achieving ecological relevance in the implementation, ensuring it makes sense to users. When referring to adaptability, the implementers of the SMH intervention say it is carried out according to the “reality criterion” of those implementing it. In other words, since the original design may not have considered the context in which the intervention was carried out, it is necessary to modify practices without altering the program’s foundations. This involves considering the experience of the school, teachers, administrators, and children who will receive the intervention. Hence, there is a particular relevance of ecological interventions (Quote 13).

An example of this is what happens in rural schools, where the program is well-received. However, due to the demands imposed by the context, implementers adapt to meet the needs of other types of psychosocial interventions (Quote 14). Additionally, understanding what can be adapted in an intervention is often recognized through trial and error.

The Reflection and Evaluation dimension was understood as both quantitative and qualitative feedback on the progress and quality of the implementation, accompanied by regular updates on progress and on personal and team experiences; implementers emphasize the importance of reflection in their professional practice. However, this reflection oscillates between formality and informality. Despite the existence of specific spaces where this type of interaction is formalized (e.g., technical meetings) (Quote 15), it generally occurs unexpectedly. Specifically, reflection is often an informal activity carried out in everyday settings, particularly during routine meetings.

Evaluation is formalized through semi-annual evaluation sessions where results are presented and team reflections on achievements take place, discussing ways to improve the program’s implementation (Quote 16). The importance of having formal and timely feedback on evaluations is emphasized; otherwise, there could be demotivation due to uncertainty and a lack of stimulus (Quote 17). Therefore, a central element would be providing feedback on various activities and reports requested within the framework of the intervention, avoiding executing teams feeling that they are performing tasks without a clear and defined purpose.

In summary, ensuring the implementation process itself requires room to adapt elements related to the program’s content or strategies without compromising its core components, together with timely and structured mechanisms for reflection and evaluation that provide implementers with a clear understanding of the intervention’s direction and purpose.

A synthesis of the main findings related to the characteristics of elements based on the analysis using the CFIR model is presented in Table 4.

## 4 Discussion

Our findings indicate that the implementation of SMH intervention in the Chilean context is influenced by a multi-level set of factors, consistent with evidence indicating that school-based implementation is shaped by factors operating at multiple levels, from internal organizational condition, such as available resources, physical spaces, and characteristics of implementers and beneficiaries; to broader external influences, including cultural norms and public policies (1, 32). Beyond these factors, the role of training and technical assistance appears decisive in supporting implementation processes (48).

In school settings, successful implementation requires alignment with existing school priorities and organizational structures, commitment from school leaders and administrators, effective communication among key stakeholders, and program adaptability to the school context. Consequently, schools face structural constraints, including limited time, staff workload, and insufficient resources, that can hinder implementation efforts. Together, these findings reinforce the view of schools not merely as settings where interventions take place, but as organizational systems that actively shape implementation processes and outcomes (2).

Regarding the elements associated with implementation conditions and their beneficiaries, our findings show that the dimensions of Readiness for Implementation and Implementation Climate are particularly relevant and sensitive to implementers.

The resources are highly relevant to Readiness for Implementation; this is a significant element in achieving program results, as reported in other studies in Chile (4, 12). Therefore, it is important to adjust the temporal, material, and human requirements for implementing an intervention (33), especially when human resources and available time are insufficient to achieve the objectives. The constant need for training on emerging topics such as migration and gender, highlighted by participants, aligns with the necessity of cultural adaptation to maintain the relevance of mental health interventions across diverse populations (22). As our results show, the demand to incorporate themes such as sexual diversity and multiculturalism reflects a proactive search for contextual fit. One possibility in this regard is that the necessary resources should be determined in advance, taking the entire community into account (35).

The Implementation Climate is one of the most complex issues to evaluate (48). The capacity for change absorption, that is, how willing implementation settings are to accept modifications is broadly relevant to implementers, with experiences varying across institutions and depending on the leadership in place (19). Our findings underscore the importance of alignment across the organizational structures involved in implementing an SMH intervention. In fact, alignment between the intervention and institutional objectives, priorities, demographics, and existing systems is necessary (42).

Concerning the elements associated with implementers, our findings indicate the leadership exercised by those coordinating an intervention appears to be a particularly important element; this could impact student performance, implementation, program outcomes, and school climate (32, 40, 42). It has been demonstrated that school leaders are key facilitators of the implementation of mental health programs and comprehensive school reform efforts (49).

The central role of leadership identified in our study aligns with the findings of Lyon and Bruns (28), which emphasize that school principals’ active participation and decision-making are essential for the systematic implementation of evidence-based practices.

For program implementers, feeling validated by leaders allows them to fully develop their work, both technically and creatively, by creating healthy, empowering workspaces. The evidence supports a continuous review of the work of leaders responsible for interventions and how they align with the required needs even before implementation (40, 50).

Regarding leadership, our study revealed that coordinators’ and school authorities’ engagement was perceived as a decisive factor in the Implementation Climate, underscoring that school leaders play a fundamental role that extends beyond mere administrative support (28). The active involvement of leaders in assessing and selecting evidence-based interventions is fundamental to ensuring the program aligns with the school’s existing systems and priorities. In this study, the preference for horizontal leadership and the need for leaders to validate the technical work of implementation teams suggest that when leadership is perceived as collaborative rather than controlling, it facilitates the buy-in necessary for the program to be regarded as a legitimate and integrated school priority rather than an external imposition (28, 42).

Our findings related to the elements associated with implementation highlight a persistent tension between fidelity to the original design and the need for local adaptation. From this perspective, adaptations represent an essential effort to reconcile the intervention’s internal logic with its external validity. While implementers often describe these modifications as reactive responses to immediate school demands, this process can be analyzed in terms of the value the intervention adds to the context in which it is implemented. That is, modifications observed in Chilean schools are not mere deviations from protocol, but rather intuitive attempts by implementers to balance the Value Equation (16).

By aligning the program with the school’s “reality criterion,” implementers are actively seeking to increase the intervention’s value and contextual fit. However, as noted in the results, the reactive nature of these changes—often occurring through trial and error—underscores the need for more proactive implementation strategies to guide adaptations while preserving the intervention’s intended functions and overall integrity (16, 18). To mitigate this risk, we argue for a shift from reactive to proactive adaptation strategies, using frameworks such as IDEA (18). This approach provides an iterative decision-making on adaptations by helping implementation teams assess whether proposed changes are likely to enhance contextual fit while preserving the intervention’s intended functions.

The literature identifies Reflection and Evaluation as central and priority axes of an intervention [e.g., Fixsen et al. and Ho et al. (51, 52)]. In our study, implementers of the SMH intervention highlighted that while reflection often occurs informally and spontaneously, evaluation is typically governed by the program’s formal design. However, the perceived lack of structured feedback remains a significant barrier.

We observed that without feedback, evaluations lose their meaning for implementers. Along with monitoring, feedback needs to be prioritized, as it is essential for responding to the implementation objective and its long-term effects (33, 53). Strategies with a more solid empirical foundation and more promising outcomes have included supervision and monitoring as part of the feedback process (53). A lack of assessment and feedback can negatively impact interventions, as SMH interventions are complex and subject to constant modifications (36). Therefore, a format is needed that allows documenting the implementation experience while being systematically assessable. This is why frequent self-reports and surveys are recommended (50).

In summary, we identified certain elements that require special attention when implementing an SMH intervention. The first of these pertain to resources and time, which need to be available and sufficient. Adequate training and access to information on program-specific topics, as well as addressing emerging issues that may require extra support from implementing teams are crucial.

A second element concerns the need for support from the entire educational community and the various stakeholders involved in the intervention to ensure its correct implementation. This aligns with the goal of increasing and encouraging the involvement of diverse stakeholders, leading to positive outcomes related to intervention fidelity and adoption (53). This implies a structural alignment among different levels of organization within SMH intervention. While the intervention is implemented at the school level, coordination across different territorial organizational levels is required for successful implementation.

Finally, we acknowledge that adaptation of both intervention content and implementation strategies remains an under-addressed dimension within SMH implementation research. Although recognizing the importance of intervention fidelity, interventions implemented in the community are exposed to requirements that demand approaches not initially anticipated, highlighting the tension between adaptability of content (e.g., the incorporation of emerging content related to multiculturalism or sexual diversity), the adaptability of strategies (e.g., changes related to scheduling, leaders’ participation, coordination among stakeholders, and available resources), and the inherent and complexity in more participatory interventions (42). Adaptations need to be recognized as plausible elements of an intervention and, as such, considered from the outset.

The interventions are unlikely to be performed without some level of modification; in fact, they constitute an inevitable part of the implementation process rather than a failure of fidelity (19, 20); especially considering that these adaptations often account for cultural relevance while ensuring the proper execution of an intervention (17, 21). Following Miller et al. (18), technical guidelines need an iterative decision-making process to determine which components require adaptation and which remain core. This highlights the need to consider adaptation as part of the implementation process, which requires not only technical guidance but also the development of judgment and skills needed to make context-specific decisions. In this sense, our results show that teams may be well trained to implement, but they develop their capacity to adapt through experience, contextual knowledge, and local decision-making, rather than through formal training.

Based on our findings, one recommendation for adapting programs to each school is to collaborate with various stakeholders in the school community and conduct formative assessments. This implies that developmental evaluation of the intervention should be conducted jointly with the school leadership team before the intervention begins (53). This would allow the team to assess and adjust the available time, physical space, and materials, as well as the school’s capacity to ensure these resources are available. It would also allow the team to identify the number and types of other psychosocial programs already operating in the school, in order to anticipate the over-intervention documented in our findings. In addition, it would allow the school leadership team to explicitly establish commitments regarding activity scheduling, teacher participation, and internal communication, as well as to identify emerging local issues that may require content adaptations, such as migration or gender diversity.

Likewise, an implementation-focused evaluation (53) should be considered, including the relationship between the planned and delivered dose, as well as the adaptations made and the rationale behind them. These considerations could help guide adaptation decisions by allowing implementers to assess whether contextual conditions are adequate before implementation begins. In addition, since the actors involved participate in decision-making, this approach would allow them to assess whether a given adaptation is consistent with the intended design of the program or intervention. This engagement facilitates the identification of the elements that implementers perceive as most acceptable and feasible within their local context. Evidence suggests that collaboratively selected components are more likely to be adopted and sustained with higher fidelity (54).

The ability to identify and safeguard certain elements when implementing a school mental health intervention needs to not only lead to achieving intervention objectives but also ensure its functionality (52). Simultaneously, implementing an intervention in the community facilitates the transferability of favorable outcomes. In this regard, given the characteristics of the program studied, the transferability of the findings may occur internally, since the program—as it is implemented throughout the country—would allow the identified elements to be observed and considered both by the implementing teams themselves and by the regional authorities responsible for its implementation, thereby promoting learning and improving its implementation. Similarly, the findings can guide other school mental health programs by providing evidence from a large-scale national program and generating knowledge that is potentially transferable to other countries in the region facing similar challenges in implementing school mental health interventions.

### 4.1 Limitations and strengths

Our study is not without limitations. While elements in the implementation process of an SMH intervention have been identified and described, it cannot be assumed that these elements are universal across all contexts (24, 52). Indeed, when working with diverse human groups in different spaces, the same intervention can yield vastly different results in another population or location. This variation may arise due to differences in context, diverse beneficiary behaviors, cultural norms restricting certain activities, or implementers choosing to omit or alter program elements due to execution difficulties, lack of relevance or low efficacy (1, 26). Additionally, the qualitative data exclusively reflects the perspectives of executing teams and coordinators. The omission of other key stakeholders—such as teachers, school principals, and parents—represents a limitation, as it restricts the understanding of factors like Acceptability and the complexities of the Inner Setting from the perspective of the final beneficiaries. Future research has to incorporate these diverse voices to achieve a more comprehensive, 360-degree assessment of the implementation context and its impact on service delivery.

Furthermore, coding reliability was addressed through independent coding followed by a consensus process, rather than through a statistical measure of inter-coder agreement. Future studies could use independent double coding to allow for the calculation of such a statistic.

Although it was not possible to use the updated version of the CFIR, the use of the original version to evaluate a national-scale school mental health program is a major strength of our study. While many studies focus on small-scale pilot interventions, this research provides empirical insights into the “real-world” determinants of implementation for the SfL program, one of the largest initiatives of its kind globally. Furthermore, the qualitative depth achieved through interviews with both implementers and coordinators enables a multi-level understanding of the barriers and facilitators that influence sustainability and contextual fit across diverse educational settings.

One notable contribution of our study is its applied nature, as it successfully identified tangible elements present in the implementation process of a national SMH intervention, recognizing the contribution of the CFIR as a program evaluation model implemented in schools (40, 41).

In future research, evaluating the impact of each of these elements on measured and expected intervention outcomes is recommended. This approach would enable more robust comparisons between studies to identify which constructs influence results and under what conditions (39).

## Data Availability

The raw data supporting the conclusions of this article will be made available by the authors, without undue reservation.
