# Supplementary material for: Safeguarding implementation elements in a national school mental health intervention: implementers’ perspectives
Source: Front Public Health. 2026 Oct 28;14:1895117. doi: 10.3389/fpubh.2026.1895117 (PMC13751449; doi:10.3389/fpubh.2026.1895117)
Supplement: Supplementary file 1 [file Data_Sheet_1.DOCX]

**Supplementary Material 1**

**Crosswalk between the original CFIR (2009) and the Updated CFIR (2022)**

For this study, the original version of the Consolidated Framework for Implementation Research (CFIR) (1) was used to guide the study design, data collection, and analyses. In the year the study began, an updated version of the CFIR was published (2), in which domains and constructs were reorganized and new ones were added. Since the interview guidelines and coding scheme were based on the original version of the CFIR, we retained its use, as modifying the framework would have compromised the consistency of the coding and analysis process.

To situate our findings within the current version of the framework, this supplementary material maps the six dimensions highlighted in this study (presented in Tables 2 and 3 of the main text) onto their corresponding constructs in the updated CFIR, along with a brief description of the changes between the two versions.

***Crosswalk for the CFIR dimensions reported in this study***

| **Dimension reported in this study**  **CFIR, 2009** | **Domain**  **CFIR, 2009** | **Corresponding construct in the Updated CFIR**  **CFIR, 2022** | **What changed** |
| --- | --- | --- | --- |
| Readiness for Implementation  (subconstructs: Leadership Engagement; Available Resources; Access to Knowledge and Information) | Inner Setting | Available Resources (with subconstructs Funding, Space, Materials & Equipment) and Access to Knowledge & Information — Domain Inner Setting.  Leadership engagement is now captured through the Individuals Domain – Roles Subdomain (High-level Leaders / Mid-level Leaders) and Characteristics Subdomain (Motivation) | Readiness for Implementation was removed as a stand-alone construct and reclassified as an antecedent assessment. Its three original subconstructs were redistributed among the remaining constructs. Resource- and information-access components remained in Inner Setting as full constructs.  Leadership engagement component was separated, renamed, and relocated in Individuals Domain: Roles Subdomain (High-Level & Mid-Level Leaders); and Characteristics Subdomain (Motivation). |
| Adaptability | Intervention Characteristics | Innovation Adaptability — Innovation Domain | Direct rename in line with the Intervention Characteristics → Innovation domain change; definition essentially unchanged (degree to which the innovation can be modified or tailored to local context). |
| Reflecting & Evaluating | Process | Reflecting & Evaluating, with two subconstructs: Implementation and Innovation — Implementation Process Domain | Retained with the same name and core definition; now explicitly split into two subconstructs distinguishing feedback about the success of the implementation process itself from feedback about the innovation's own performance. |
| Implementation Climate  (subconstructs: Tension for Change; Compatibility; Relative Priority; Organizational Incentives and Rewards; Goals and Feedback; Learning Climate) | Inner Setting | Tension for Change; Compatibility; Relative Priority; Incentive Systems; Mission Alignment — all promoted to full Inner Setting constructs.  Learning Climate was relocated under Culture as Learning-Centeredness. | Implementation Climate was removed as a parent construct and reclassified as an antecedent assessment. Five of its former subconstructs became independent Inner Setting constructs (Organizational Incentives & Rewards were renamed Incentive Systems, and Mission Alignment was added, partly absorbing Goals and Feedback). Learning Climate moved to the Culture construct. |
| Needs and Resources of Individuals Served by the Organization  (cited in-text as "Patient Needs and Resources") | Outer Setting | Construct separated and relocated:  Innovation Recipients, in Role’s subdomain; and Need in Characteristics Subdomain — Individual Domain  Recipient-Centeredness is part of Culture— Inner Setting Domain. | This was one of the most substantial relocations in the update: “needs” moved from an organizational-level Outer Setting construct to an individual-level Characteristics construct, applied to Innovation Recipients. This reflects the CFIR 2.0 goal of centering innovation recipients rather than treating their needs as an attribute of the serving organization. |
| External Policy and Incentives | Outer Setting | Policies & Laws; Financing; External Pressure (with subconstructs Societal Pressure, Market Pressure, Performance-Measurement Pressure) — Outer Setting | The single broad 2009 construct was disaggregated into three more granular Outer Setting constructs, separating regulatory/legal drivers, funding mechanisms, and different sources of external pressure. |

This crosswalk was developed based on the findings of this study. Claude (Anthropic, 2026) was used to support this process, drawing on the original CFIR publication (1), the CFIR update (2), and the official construct definitions available at cfirguide.org. All mappings were reviewed and verified by the authors against these primary sources.

1. Damschroder LJ, Aron DC, Keith RE, Kirsh SR, Alexander JA, Lowery JC. Fostering Implementation of Health Services Research Findings into Practice: A Consolidated Framework for Advancing Implementation Science. Implement Sci. 2009; 4:50. doi:10.1186/1748-5908-4-50
2. Damschroder LJ, Reardon CM, Widerquist MAO, Lowery J. The updated Consolidated Framework for Implementation Research based on user feedback. Implement Sci. 2022;17(1):75. doi:10.1186/s13012-022-01245-0
