# Supplementary material for: Safeguarding implementation elements in a national school mental health intervention: implementers’ perspectives
Source: Front Public Health. 2026 Oct 28;14:1895117. doi: 10.3389/fpubh.2026.1895117 (PMC13751449; doi:10.3389/fpubh.2026.1895117)
Supplement: Supplementary file 2 [file Data_Sheet_2.DOCX]

**Supplementary Material 2**

**Interview Guide**

Intervention Characteristics

Questions considered in the interviews: What are the characteristics and objectives of the program?

- Is the intervention internal or external to the school? (Dimension: Source of the Intervention)
- Does the intervention have a theoretical or practical foundation? (Dimension: Strength and Quality of the Evidence)
- Does the intervention offer any advantages or differences compared to other interventions in the school? (Dimension: Relative Advantage)
- How is the implementation of the workshops carried out concerning to the original plan? Does it need to be adapted? (Dimension: Adaptability)

Outer Setting

Questions considered in the interviews: What characteristics does the external context in which the intervention is developed have?

- Are the needs of the educational community clear to the implementing entity? What facilitators and barriers influence the impact of the intervention on these needs? (Dimension: Needs and Resources of the Individuals Served by the Organization)
- What networks do the coordination and implementing team manage to develop with other community institutions? (Dimension: Cosmopolitanism)
- Are there requirements for implementation at specific times or in different forms? (Dimension: Peer Pressure)
- Are there external guidelines and/or rewards for implementing the workshops? (Dimension: External Policy and Incentives)
- Is the intervention compatible with the school's times, objectives, and ways of working? (Dimension: Networks and Communications)

Inner Setting

Questions considered in the interviews: What characteristics does the internal context in which the intervention is developed have?

- What forms and means of communication are used for the coordination of this structure? What aspects are identified as facilitators and obstacles to communication? (Dimension: Networks and Communications)
- Is the intervention compatible with the implementing team's times and ways of working and coordination? Are team members encouraged to participate in the implementation? Are there support and feedback instances? (Dimension: Implementation Climate)
- How would you define the commitment of the leaders to the implementation? What resources—financial, physical, material, human, and time—are available? (Dimension: Readiness for Implementation)

Characteristics of the Individuals

Questions considered in the interviews: What characteristics of the participants facilitate or hinder the implementation of the intervention?

- What tools and knowledge do the implementing teams have to carry out the workshops? (Dimension: Self-Efficacy)
- What motivates you and the team to do this work? (Dimension: Individual Identification with the Organization)
- What personal characteristics have facilitated or hindered your work? (Dimension: Other Personal Attributes)

Implementation Process

Questions considered in the interviews: Who are the main actors of the intervention? Who is most impacted, and how?

- How do you plan the implementation of preventive workshops? (Dimension: Planning)
- Who has influenced, positively or negatively, the beliefs and commitment of the actors regarding the intervention? What strategies are used to engage the educational community? Are there individuals, institutions, or external programs that have shown commitment to the implementation? (Dimension: Engagement)
- What aspects facilitate or hinder the implementation according to the plan? (Dimension: Execution)
- How and when is the process evaluated? (Dimension: Reflection and Evaluation
