# Supplementary material for: Safeguarding implementation elements in a national school mental health intervention: implementers’ perspectives
Source: Front Public Health. 2026 Oct 28;14:1895117. doi: 10.3389/fpubh.2026.1895117 (PMC13751449; doi:10.3389/fpubh.2026.1895117)
Supplement: Supplementary file 3 [file Data_Sheet_3.DOCX]

**Supplementary Material 3**

**Details on the number of citations by domain**

| **Dimension**  **(according to CFIR model)** | **Number of Citations** | **Relative Frequency (%)** | **Cumulative Frequency (%)** |
| --- | --- | --- | --- |
| Readiness for implementation | 192 | 13.24 | 13.24 |
| Adaptability | 150 | 10.34 | 23.58 |
| Reflecting & evaluating | 120 | 8.28 | 31.86 |
| Implementation climate | 116 | 8.00 | 39.86 |
| Patient needs and resources | 87 | 6.00 | 45.86 |
| External policy and incentives | 85 | 5.86 | 51.72 |
| Networks & communication | 77 | 5.31 | 57.03 |
| Knowledge and beliefs about intervention | 74 | 5.10 | 62.13 |
| Engaging | 69 | 4.76 | 66.89 |
| Self-Efficacy | 64 | 4.41 | 71.30 |
| Cosmopolitanism | 62 | 4.28 | 75.58 |
| Culture | 61 | 4.21 | 79.79 |
| Structural characteristics | 59 | 4.10 | 83.69 |
| Planning | 57 | 3.90 | 87.79 |
| Evidence, strength, quality | 47 | 3.24 | 91.03 |
| Relative advantage | 38 | 2.62 | 93.65 |
| Individual stage of change | 35 | 2.41 | 96.06 |
| Complexity | 23 | 1.59 | 97.65 |
| Triability | 17 | 1.17 | 98.82 |
| Peer pressure | 12 | 0.83 | 99.65 |
| Design quality and packaging | 5 | 0.34 | 99.99 |
